# Using cryoEM and cryoET to visualize membrane penetration of a non-enveloped virus

**DOI:** 10.1016/j.xpro.2022.101825

**Published:** 2022-11-11

**Authors:** Xian Xia, Z. Hong Zhou

**Affiliations:** 1 Department of Microbiology, Immunology & Molecular Genetics, University of California, Los Angeles, Los Angeles, CA, 90095, USA; 2 California NanoSystems Institute, University of California, Los Angeles, Los Angeles, CA, 90095, USA

**Keywords:** Microbiology, Microscopy, Structural biology, Cryo-EM

## Abstract

Key to cell entry by non-enveloped viruses is virus-cell interactions at the cell or endosomal membrane. Here, we detail our protocols to capture such interactions between non-enveloped virus bluetongue virus (BTV) and vesicular membrane by cryogenic electron microscopy (cryoEM) and tomography (cryoET). Key steps include virus isolation, liposome preparation, virus-liposome incubation and vitrification, cryoEM and cryoET imaging, data processing for 3D reconstruction, and subtomogram averaging. The protocols can be generally applicable to studies of cell entry by other non-enveloped viruses.

For complete details on the use and execution of this protocol, please refer to Xia et al. (2021).

## Highlights

• Purification of intact BTV virions from infected BHK-21 cells
• Low pH induced conformational change of BTV on cryoEM grid
• Atomic-resolution structures of BTV at different states by single-particle cryoEM
• Capturing membrane penetration of BTV by cryoET and subtomogram averaging

## Abstract (editor highlights)

Publisher’s note: Undertaking any experimental protocol requires adherence to local institutional guidelines for laboratory safety and ethics.

## Graphical abstract

**Figure:**
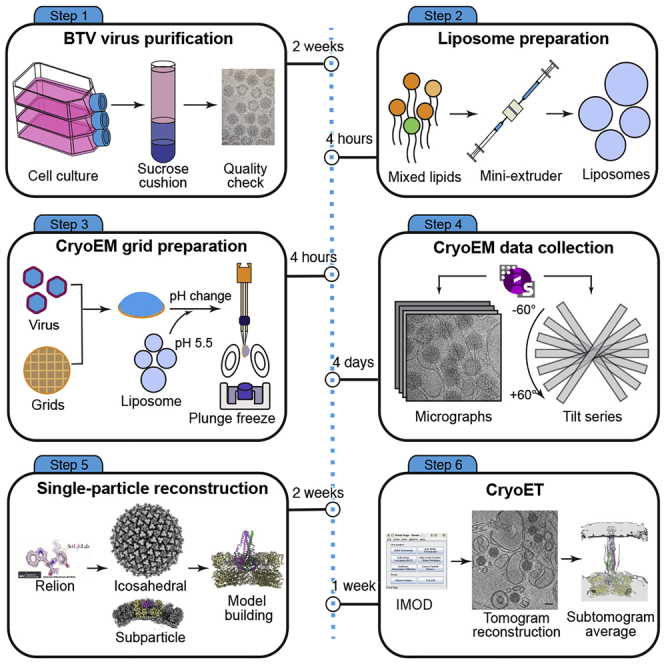

## Before you begin

### Overview

Bluetongue virus (BTV), a non-enveloped dsRNA virus of the Reoviridae and a significant threat to livestock, enters host cells via the endocytic pathway. The late endosome’s low pH triggers conformational changes of the BTV membrane penetration protein VP5 and primes the virus to interact with the endosomal membrane for viral escape to the cytosol from the endosome. Low pH-triggered BTV conformational changes and membrane penetration process are both highly dynamic. The protocols below describe the detailed steps we employed to capture the dynamic structures and membrane penetration process, including cell culture, virus isolation, cryogenic electron microscopy (cryoEM) sample preparation, imaging, single-particle reconstruction, cryogenic electron tomography (cryoET) tilt series acquisition, and subtomogram averaging. BTVvirions are purified from baby hamster kidney fibroblast cells (BHK-21) and incubated with liposomes at low pH on cryoEM grids to mimic the condition of the late endosome. By optimizing incubation time before plunge-freezing the grids, we captured various intermediate states of BTV interacting with vesicular membrane.

### Prepare workspace and materials

**Timing: 15 min**

1. Designate workspace with certification of biosafety level 2 for BTV from the environmental health and safety department of your institute. All materials with BTV or BTV-infected cells should be handled in a biosafety cabinet following proper biosafety protocols.

**CRITICAL:** Make sure to follow your institute’s approved biosafety protocols. Liquid waste should be disinfected with 10% of bleach and solid waste disposed according to your facility’s regulation. Clean the surface of your workspace with 70% of ethanol upon completion of your work.

2. Set the incubator to 35°C (favorite temperature of BTV growth) with 5% CO_2_.
3. Set the water bath to 35°C and pre-warm a bottle of Dulbecco’s Modified Eagle Medium (DMEM) in the water bath.

### BHK-21 cell culture

**Timing: 1 week**

4. Thaw a vial of cryopreserved BHK-21 cell by gentle agitation at 35°C in the water bath. Thawing should be rapid: less than 2 min or until ice is melted.
5. Decontaminate the vial with 70% ethanol, transfer the cells into a T75 flask in the biosafety cabinet and slowly add 14 mL DMEM medium supplemented with 10% fetal bovine serum and antibiotics (penicillin at 100 U/mL and streptomycin at 100 μg/mL).
6. Shake the flask gently, separating the cells thoroughly to avoid aggregation, and set the flask in the incubator at 35°C with 5% CO_2_.
7. After 4–6 h, examine the cells by an inverted light microscope. If most of the cells attach to the bottom of the flask, replace the medium in the flask with 15 mL of fresh medium.

***Note:*** Living cells are flat and polygonal in shape and attach to the bottom of the flask, while dead cells are round and float in the medium. The viability of the cells is usually higher than 80%. If most of the cells are dead, then check the condition of the frozen cell.

8. Keep the flask in the incubator for 2 days until the cells reach 90% confluence before subculturing into a T175 flask.
9. Remove and discard the medium in the flask.
10. Rinse the cell gently with 10 mL PBS to remove all traces of serum which inhibits trypsin.
11. Discard the PBS and add 3 mL of trypsin-EDTA solution to the flask.
12. Keep the flask in the incubator until cell layer is detached.

**CRITICAL:** Observe the flask under the microscope frequently to prevent over digestion which will damage the cells; digestion occurs usually within 5–10 min. If the trypsin digestion is too fast, then slow this process by either diluting the trypsin-EDTA solution or keeping the digestion at 25°C.

13. Add 7 mL fresh medium to stop the digestion and pipette gently to disperse the cells.
14. Add 2 mL (1:5 subcultivation) of the cell suspension and 33 mL fresh medium to each new T175 flask.

***Note:*** A subcultivation ratio of 1:3 to 1:10 is recommended.

15. Continue scaling up the cell culture (repeat steps 8–14) until you have 10 T175 flasks of cells at 90% confluence.

## Key resources table

**Table:** 

REAGENT or RESOURCE	SOURCE	IDENTIFIER
**Bacterial and virus strains**		

BTV-1	Maintained in the lab	N/A

**Experimental models: Cell lines**		

BHK-21	ATCC	CCL-10

**Chemicals, peptides, and recombinant proteins**		

Tris base	Sigma-Aldrich	Cat# 93362
HEPES	Sigma-Aldrich	Cat# 54457
Sodium chloride	Sigma-Aldrich	Cat# S6546
Sodium citrate	Sigma-Aldrich	Cat# 71402
Chloroform	Sigma-Aldrich	Cat# 366927
NP-40	Sigma-Aldrich	Cat# I8896
Sucrose	Sigma-Aldrich	Cat# S7903
N-Lauroylsarcosine sodium salt	Sigma-Aldrich	Cat# L9150
Phosphate buffer saline (PBS)	Gibco	Cat# 10010031
cOmplete Protease Inhibitor	Roche	Cat# 11836170001
DMEM	Sigma	Cat# D6429
Fetal bovine serum (FBS)	R&D Systems brand	Cat# S11150H
Penicillin/Streptomycin(100×)	HyClone	Cat# SV30010
Trypsin-EDTA (10×)	Gibco	Cat# 15400054
DOPC	Avanti	Cat# 850375
DOPE	Avanti	Cat# 850725
LBPA	Avanti	Cat# 857133
Cholesterol	Avanti	Cat# 700000
Gold beads	Cell Microscopy Center	Cat# PAG10nm/S
Uranyl acetate	Electron Microscopy Sciences	Cat# 22400

**Software and algorithms**		

SerialEM	(Mastronarde, 2005)	https://bio3d.colorado.edu/SerialEM/
MotionCor2	(Zheng et al., 2017)	https://msg.ucsf.edu/software
ETHAN	(Kivioja et al., 2000)	https://www.sciencedirect.com/science/article/pii/S1047847700942795
Relion 3.1	(Scheres, 2016)	https://www3.mrc-lmb.cam.ac.uk/relion/
Local reconstruction script	(Ilca et al., 2015)	https://github.com/OPIC-Oxford/localrec/wiki
UCSF ChimeraX	(Pettersen et al., 2004)	https://www.cgl.ucsf.edu/chimerax
COOT	(Emsley and Cowtan, 2004)	https://www2.mrc-lmb.cam.ac.uk/personal/pemsley/coot
PHENIX	(Adams et al., 2010)	https://phenix-online.org/
IMOD	(Kremer et al., 1996)	https://bio3d.colorado.edu/imod/
PEET	(Nicastro et al., 2006)	https://bio3d.colorado.edu/PEET/

**Deposited data**		

CryoEM map of IMS1	(Xia et al., 2021)	EMD-24686
CryoEM map of IMS2	(Xia et al., 2021)	EMD-24685
CryoEM map of IMS3	(Xia et al., 2021)	EMD-24687
CryoEM map of low-pH state	(Xia et al., 2021)	EMD-24684
Atomic model of IMS2	(Xia et al., 2021)	PDB: 7RTO
Atomic model of low-pH state	(Xia et al., 2021)	PDB: 7RTN

**Others**		

Biosafety cabinet	NuAire	N/A
CO_2_ cell culture incubator	NuAire	N/A
Cell culture flasks	Corning	Cat# CLS431306-84EA
Cell scraper	Corning	Cat# 07-200-365
Thinwall ultra-clear tube (SW28)	Beckman	Cat# 344058
Thinwall ultra-clear tube (SW41)	Beckman	Cat# 344059
Mini-Extruder set	Avanti	Cat# 610000
PC Membranes 0.2um	Avanti	Cat# 610006
Glass syringe	Hamilton	Cat# EW-07938
PELCOeasiGlow glow discharge cleaning system	Ted Pella	Cat# 91000S
Ultrathin carbon on Lacey Carbon support film	Ted Pella	Cat# 01824
Blotting paper, Grade 595	Ted Pella	Cat# 47000-100
Cryo-sample plunger	Thermo Fisher Scientific	Vitrobot Mark IV
200 kV TF20 High-Resolution EM	FEI	TF20
300 kV Titan Krios High-Resolution EM	FEI	Titan Krios

## Materials and equipment

**Table:** Lysis Buffer

Reagent	Final concentration	Amount
1 M Tris-HCl, pH 8.8	100 mM	5 mL
5 M NaCl	50 mM	0.5 mL
10% NP40	0.1%	0.5 mL
protease inhibitor cocktail (50×)	1×	1 mL
ddH_2_O		43 mL
Total volume		50 mL

***Note:*** pH 8.8 is used to keep the high pH conformation of BTV. Otherwise, the virus is not stable during purification. Prepare fresh buffer for each purification or store the buffer without protease inhibitor at 4°C and add protease inhibitors prior to use.

**Table:** 66% (w/w) sucrose buffer

Reagent	Final concentration	Amount
1 M Tris-HCl, pH 8.8	20 mM	1 mL
5 M NaCl	50 mM	0.5 mL
10% NP40	0.2%	1 mL
sucrose	66% (w/w)	33 g
ddH_2_O		14.5 mL
Total volume		∼43 mL

***Note:*** Store at 4°C.

**Table:** 50% (w/v) sucrose buffer

Reagent	Final concentration	Amount
1 M Tris-HCl, pH 8.8	20 mM	1 mL
5 M NaCl	50 mM	0.5 mL
10% NP40	0.2%	1 mL
sucrose	50% (w/v)	25 g
Total volume		50 mL

***Note:*** Store at 4°C.

**Table:** Liposome Buffer

Reagent	Final concentration	Amount
1 M HEPES pH 7.5	10 mM	0.5 mL
5 M NaCl	100 mM	1 mL
1 M Sodium citrate pH 7.5	50 mM	2.5 mL
ddH_2_O		46 mL
Total volume		50 mL

***Note:*** Store at 4°C.

## Step-by-step method details

### Purification of BTV from infected cells

**Timing: 1 day**

This section describes the detailed protocol for BTV purification from infected BHK-21 cells. BTV is a non-enveloped virus with three layers of capsid proteins: VP2 and VP5 on the outer layer, VP7 in the intermediate layer and VP3 in the inner layer. The infectious virion, which contains all three layers of capsid proteins, is unstable and prone to losing the outer layer proteins during purification. For this reason, the purification steps here are optimized to keep the virion intact.

1. 10 T175 flasks of BHK-21 cells are maintained till 90% confluence. Change the medium with 35 mL fresh DMEM with FBS and antibiotics (penicillin at 100 U/mL and streptomycin at 100 μg/mL).
2. Add 5 mL of BTV stocks into each flask. Shake the flasks gently to mix the virus.

***Note:*** BTV1 stocks are prepared from the medium of BHK-21 cells 72 h post infection and maintained in the lab. Alternatively, BTV10 from ATCC (VR-187) can also be used. Aliquot the medium containing the virus and store at −80°C. Frequent freezing and thawing of the stock will reduce the infectivity of BTV. Among the same batch of virus, infectivity is similar. For different batches of virus stock, the volume added into each flask must be optimized.

***Alternatives:*** The titer of virus stock can be measured by a plaque assay (Baer and Kehn-Hall, 2014). The titer of virus we used is around 1.5× 10^5^/mL. Use the same multiplicity of infection (MOI) each time (about 0.03 MOI).

3. Set the flasks in the incubator for about 70 h. Observe the infected cells frequently 48 h post infection. Infected cells will change to a round shape in comparison to the polygonal shape of uninfected cells. The ratio of infected cells will increase over time. About 70 h post infection, the ratio reaches 80%, at which point cells should be harvested.
4. Discard the media and add 1.5 mL pre-chilled lysis buffer into each flask. Collect the cells by using a cell scraper. Combine all cells into a 50 mL tube and add lysis buffer to reach a total volume of 20 mL.
5. Pipet the sample up and down several times to separate the cells. Keep the sample on ice for 15 min.
6. Centrifuge the resulting cell lysate at 2,000 × *g* for 10 min to pellet cell nuclei.
7. Load the supernatant, which contains intact virions released from the cells, onto a double cushion with 4 mL 66% (w/w) sucrose buffer on the bottom and 10 mL 50% (w/v) sucrose buffer on the top.
8. Centrifuge the sample at 100,000×*g* using an SW28 rotor for 1 h at 4°C.
9. Remove the sample above the virus band and collect the band at the interface of the two sucrose cushions and dilute 10 times to 10 mL in 20 mMTris-HCl (pH 8.8).

**Figure 1:**
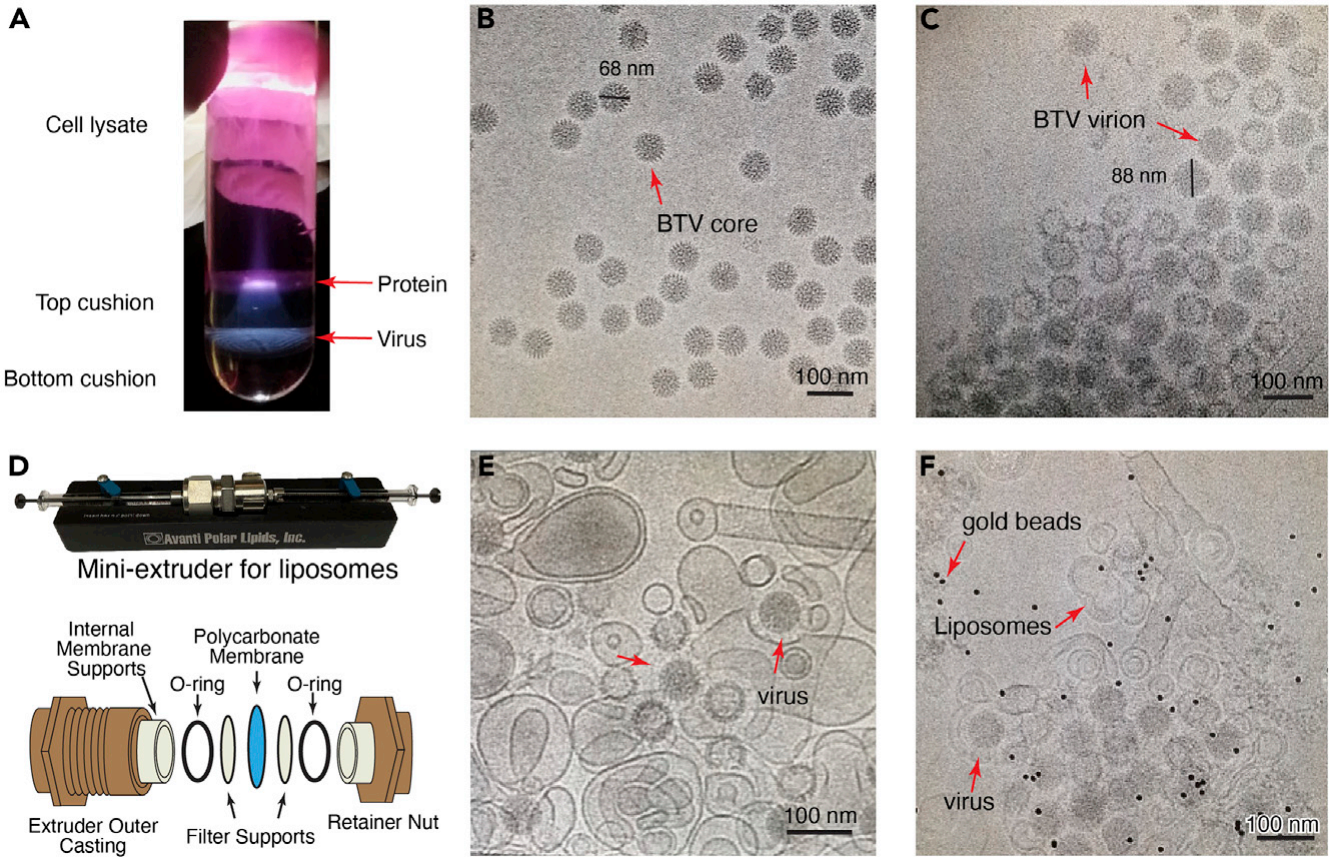
Virus purification and cryoEM grid preparation (A) Image of the sucrose cushion after centrifugation. Position of the protein debris and virus band are indicated by red arrows. (B and C) CryoEM images of purified virus. BTV core (B) is easily differentiated from BTVvirion (C) by measuring the particle diameter. (D) Mini extruder used for liposome preparation. The hole size of the polycarbonate membrane is 200 nm. (E and F) CryoEM images showing the interaction of BTV with liposome membrane at low pH. Grids with 10 nm gold beads are used for cryoET.

***Note:*** Use a flashlight to direct the position detection of the band (Figure 1A). Carefully remove the sample above the band and collect the band with a 1 mL pipet. The collected volume of the band should be less than 1 mL to avoid high concentration of sucrose in the sample after 10× dilution. If the collected volume is more that 1 mL, then dilute the sample to 20 mL and use 2 tubes to pellet the virus in step 12.

10. Pipet the sample several times to separate the virus aggregation after adding N-Lauroylsarcosine sodium salt to a final concentration of 0.1% and incubate on ice for 10 min.

***Note:*** N-Lauroylsarcosine is a detergent used to reduce virus aggregation. The final concentration of N-Lauroylsarcosine, sample incubation time and temperature are optimized.

11. Clarify the sample by centrifugation at 16,000 × *g* for 10 min.
12. Add 1 mL 30% (w/v) sucrose to the bottom of a new SW41 tube, slowly layer the supernatant from step 11 on the top of the 30% sucrose and pellet the sample at 80,000 × *g* for 1 h at 4°C in an SW41 Ti rotor.
13. Carefully remove the supernatant and resuspend the pellet in 20 μL of 20 mMTris-HCl pH 8.8.

***Note:*** Outer layer proteins of BTV (VP2 and VP5) are prone to detaching from the capsid, resulting in virus core particles as shown in Figure 1B. Check the quality of the purified virus through negative stain and cryoEM. Intact virions of BTV (Figure 1C) differ from core particles (diameter of virion is 88 nm while diameter of core particle is 68 nm). Concentration of the virus is estimated by EM. The concentration is sufficient if there are more than 5 particles in each image.

### Liposome preparation

**Timing: 4 h**

This section describes the detailed protocol for preparing liposomes which mimic the membrane of the late endosome. Lipid compositions are selected according to the composition of the late endosomal membrane. Lysobisphosphatidic acid (LBPA) is a lipid specific to the late endosome and crucial for BTV membrane penetration.

14. Prepare lipids for cryoEM from 1,2-dioleoyl-sn-glycero-3-phosphocholine (DOPC), 1,2-dioleoyl-sn-glycero-3-phosphoethanolamine (DOPE), cholesterol and lysobisphosphatidic acid (LBPA) in a molar ratio of 50:20:15:15.
15. Dissolve all lipids in chloroform at concentration of 25 mg/mL. The four lipids (DOPC 100 μL, DOPE 40 μL, cholesterol 30 μL and LBPA 30 μL) are mixed in a glass vial using glass syringes.
16. Slowly evaporate the chloroform under a nitrogen gas flow in a laboratory fume hood. Remaining traces of the solvent are removed by placing the glass vial in a vacuum desiccator for 3 h.
17. Hydrate the dried lipids in 250 μL liposome buffer. After pipetting for several times, transfer sample to a 1.5 mL Eppendorf tube.
18. Freeze-thaw the mixture 5 times in liquid nitrogen and then extrude it 21 times through a 0.2 μm polycarbonate membrane filter by using a Mini Extruder (Figure 1D).

***Note:*** Different sizes of the polycarbonate membrane filter can be used to generate different sizes of liposomes.

19. Store liposomes at 4°C and use within one week.

### CryoEM sample preparation and data collection

**Timing: 2–4 days**

This section describes the detailed protocol for cryoEM grids preparation and data collection for both cryoEM and cryoET. Briefly, the purified virus is applied to a thin continuous carbon film coated lacey grid, followed by incubation with liposomes at pH 5.5 on the grid. The grid is then blotted and plunge frozen in liquid ethane. To investigate the interaction between BTV and liposomes, both methods of cryoEM and cryoET are used. By cryoEM and single-particle reconstruction, high resolution structure of BTV can provide detailed information of conformational changes at low pH. By cryoET, we can observe the direct interactions between the long flexible stalk of BTV with the liposomal membrane.

20. Glow discharge the thin continuous carbon film coated lacey grids in an easiGlow machine for 1 min.
21. Add sodium citrate buffer (pH 5.5) to final concentration of 100 mM to titrate the liposome to pH 5.5.
22. Apply 2 μL of the purified BTVvirion to a glow discharged grid and incubate for 1 min.
23. Blot the grid manually with a filter paper to remove most of the virus and add 2.5 μL of the acidified liposomes to the grid.
24. Mount the grid into the Vitrobot. After incubation for another 30 s, blot the grid for 20 s at 8°C with 15 blot force and 100% humidity and plunge-freeze in liquid ethane by using a Vitrobot Mark IV.

***Note:*** The Vitrobot is turned on and the temperature and humidity are set to 8°C and 100%, respectively. Wait for the saturation of blot paper for 20 min prior to use. Virus will have conformational changes at low pH and easy to form aggregation. It is critical to apply virus to the grid before adding liposomes.

***Note:*** For grids of cryoET at low pH (pH 5.5), prepare grids in the same way except for mixing the liposomes with 10 nm gold beads before being applied to the grid (Figures1E and 1F).

25. Check the quality of the grid by a TF20 microscope. Freezing conditions need to be optimized if the grids are not of high quality.

***Note:*** Quality of the grid is estimated from aspects of ice thickness, virus concentration, and interaction between the viruses and the liposomes. If the ice on the grid is too thick, increase the blot time and blot force. If virus concentration is low, you need to purify the sample again (repeat steps 1–13). If no interactions between the virus and liposomes are observed, incubate the virus and liposome for a longer time in step 24.

**Pausepoint:** Freeze the rest of the virus sample with the same condition. The grids can be stored in liquid nitrogen for a long time before data collection.

**Figure 2:**
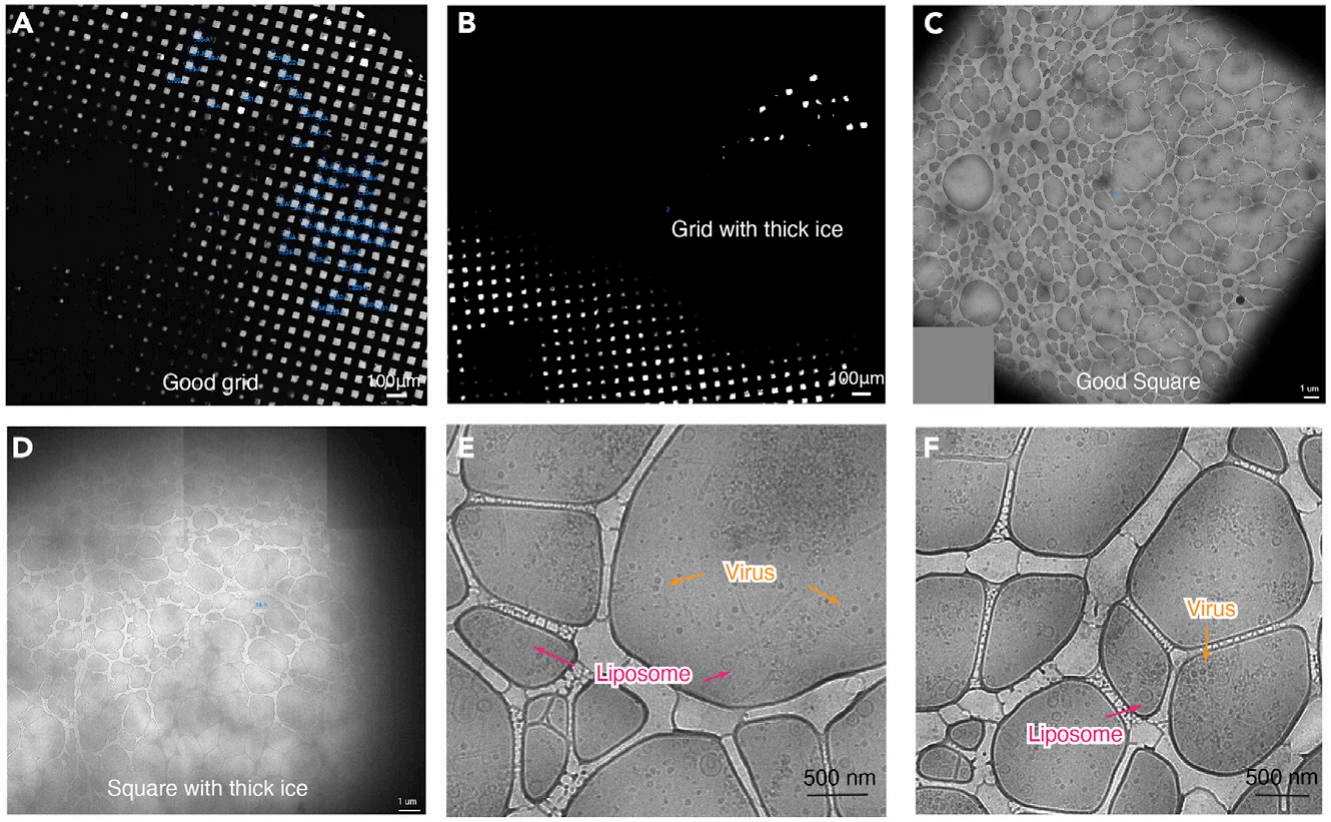
Data collection process on Titan Krios (A) Low magnification map of the grid used for data collection. Blue boxes in the image indicate squares with proper ice thickness selected for data collection. (B) Low magnification map of a grid with thick ice. (C) A typical example of a square used for data collection. (D) An example of a square with thick ice, suboptimal for data collection. (E and F) Images of the target position in view mode. Viruses and liposomes are visible at this magnification.

26. Load the optimized grids to a Titan Krios electron microscope equipped with a Gatan imaging filter (GIF) Quantum LS and a Gatan K2 Summit direct electron detector. The microscope is operated at 300 kV in super-resolution mode.
27. Align the microscope carefully (Herzik, 2021) and set up the data collection condition in SerialEM (Mastronarde, 2005).
28. Create a full map of the grid at magnification of 135× in search mode (Figures 2A and 2B). Select the squares with proper ice thickness and take a medium magnification map at 4800× for each square. In the medium magnification map, virus particles are visible (Figures 2C–2F).

**CRITICAL:** Ice thickness of the area used in data collocation is one of the key factors that determines the quality of the collected images. Images from area of thick ice (Figures 2B and 2D) have low contrast and cannot be used for high-resolution reconstruction. Ice thickness of the grid can be reduced by increase the blot time and blot force at step 24.

29. Record movies at high magnification on the selected targets which contain virus particles.
30. For single-particle reconstruction, movies are recorded at a nominal magnification of 105,000×, corresponding to a calibrated pixel size of 0.68 Å on the specimen level in super-resolution mode. Defocus is set to -1.8 to -2.6 μm. The total exposure time for each movie is set to 8 s, fractionated equally into 40 frames, leading to a total dosage of 40–50 electrons/Å^2^ on the specimen.
31. For cryoET, tilt series are collected at a nominal magnification of 53,000×, corresponding to a calibrated pixel size of 1.276 Å on the specimen level in super-resolution mode. Tilt series between −60° and +60° with a tilt increment of 3° are acquired at selected positions using a grouped dose-symmetric tilt scheme in serialEM. A movie of 8–10 frames is recorded at each tilt angle and the cumulative dosage on the specimen for a tilt series is about 120 electrons/Å^2^. Defocus is set to -3 to -4.5 μm.

***Note:*** If you collect data in a microscope with different configurations, you may have to use different microscope settings. Also, as the K3 camera has better performance and is faster than the K2, exposure time can be decreased when using a K3 camera. Please work with your facility manager to determine the microscope settings.

### Single-particle cryoEM reconstruction

**Timing: 1–2 weeks**

**Figure 3:**
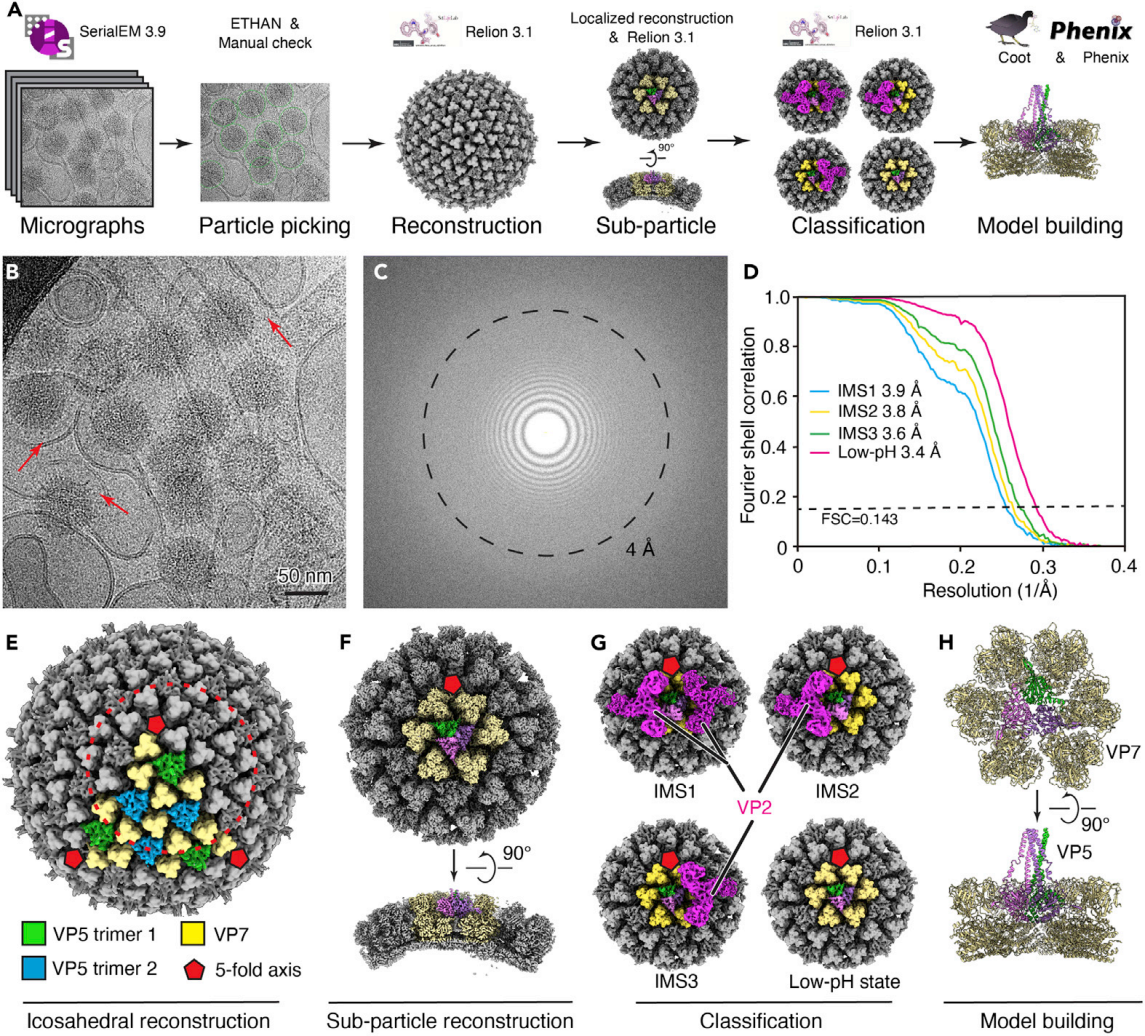
Single-particle reconstruction of the collected data set (A) Workflow of the single-particle reconstruction used in this paper. Programs used in each step are indicated. (B) A representative cryoEM image of BTV at low pH. Red arrows indicate the VP5 stalks formed after change in pH. (C) Fourier transform of the image in (A). The dashed circle shows the position of the thon ring with the best resolution. (D) Gold-standard Fourier shell correlation (FSC) coefficients as a function of spatial frequency for the sub-particle reconstructions of BTV in different states. (E) Icosahedral reconstruction of BTV at low pH. The dashed circle shows the area of sub-particle reconstruction centered on VP5trimer 1. (F) Sub-particle reconstruction of BTV by using the parameters from the icosahedral reconstruction. (G) Four different states of BTV based on the attached VP2. (H) Atomic model of VP5 and VP7 at low pH state shown in ribbon.

This section describes the detailed protocol for single-particle reconstruction and sub-particle reconstruction of BTV virus (Figure 3A). Icosahedral-reconstruction-guided sub-particle reconstruction is necessary for this data set to get high resolution structures of BTV in different intermediate states at low pH. After particle picking and extraction, the 3D reconstruction can be divided into four steps as detailed below.

32. Each movie is motion-corrected (Figure 3B) and binned 2× to a pixel size of 1.36 Å by MotionCorr2 (Zhenget al., 2017), yielding two micrographs: a dose-unweighted micrograph (used for manually screening, particle picking and defocus determination) and a dose-weighted micrograph (for particles extraction and final reconstructions).
33. In our case, 3,609 good images are selected from a total number of 3,961 through manual screening.

***Note:*** For virus with icosahedral symmetry, 10,000–20,000 virus particles are sufficient for atomic resolution reconstruction if the images collected are of high quality. How many images you need is dependent on the virus concentration.

34. Determine the defocus values of the micrographs by CTFFIND4 (Figure 3C) (Rohou and Grigorieff, 2015).
35. Pick the virus particles by ETHAN (Kiviojaet al., 2000) and then manually check to add the particles near the edges of image.

***Note:*** ETHAN is a particle picking software specific for spherical particles. The only parameter needed for particle picking is the particle radius in pixels.

***Alternatives:*** Topaz, a neuron network-based software also works for particle picking. To use Topaz, several micrographs are manually picked, and the resulting particle coordinates are used for neuron network training. Subsequently, all the micrographs will be able to be picked by using the trained model.

36. A total number of 27,138 virus particles are extracted with a box size of 768× 768 pixels and further binned to 384× 384 pixels (2.72 Å/pixel) to speed up data processing in Relion 3.1 (Scheres, 2012,^2016^).
37. In the first step of 3D reconstruction, the overall structure of BTV is reconstructed by icosahedral symmetry.
  a. Subject the particles from step 36 into 2D classifications in Relion. Classes with high resolution features (some α helices are visible in 2D average) are selected.
  b. Particles selected from 2D classification are subjected to 3D classification (K=4) by using icosahedral symmetry.
  c. 15,224 good particles are selected and subjected to a 3D auto-refinement with icosahedral symmetry yielding a reconstruction with a resolution of 5.44 Å (Figure 3E).
  d. Re-extract the particles with a box size of 768×768 pixels (1.36 Å/pixel) by using the accurate center and orientation parameters. These viral particle images and their corresponding icosahedral-related data STAR files are used in the next step.

38. In the second step of 3D reconstruction, an icosahedral-reconstruction-guided sub-particle reconstruction workflow is used to obtain near-atomic resolution structures of VP5 at low pH (pH 5.5) (Figure 3F).
  ***Note:*** In the icosahedral reconstruction, there are two VP5trimers (VP5trimer 1 and VP5trimer 2) in each asymmetric unit (Figure 3E). We used a sub-particle reconstruction method (He et al., 2019; Ilcaet al., 2015; Liu et al., 2019) centering on the first of the two VP5trimers. Due to the unsynchronized nature of conformational changes of different VP5trimers, even on the same BTV particle, this strategy of sub-particle reconstruction is essential for high resolution reconstruction and for different intermediate states of BTV at low pH.
  a. Define the position of VP5 in the icosahedral reconstruction by using UCSF Chimera volume tracer.
  b. Generate the STAR file of sub-particles by using localized reconstruction (Ilcaet al., 2015). STAR file of icosahedral reconstruction in step 37 with 15,224 virus particles is expanded by I3 symmetry (by using relion command *relion_particle_symmetry_expand*)—sixty entries with different orientations for one virus particle.
  c. A total of 913,440 sub-particles are then extracted with a box size of 300× 300 pixels from those viral particle images.
  d. Generate an initial model form the extracted particles by using relion command *relion_reconstruct.*
  e. Subjected the extracted particles to local refinement in Relion with map from step 38d as initial model, yielding a reconstruction with resolution of 3.2 Å. The VP2 density is visible at a lower threshold, indicating non-uniform occupancy of VP2 across different virions and/or even on the same virion.
39. In the third step of our workflow, focused 3D classification without orientation search is carried out in Relion (Scheres, 2012,^2016^).
  a. Generate a cylinder mask centered on VP5trimer 1.
  b. Run 3D classification with skip align option in Relion by asking for 5 classes. Four different states of BTV are obtained with 66,934 particles in intermediate state 1 (IMS1), 103,469 particles in intermediate state 2 (IMS2), 120,974 particles in intermediate state 3 (IMS3) and 572,331 particles in low-pH state (Figure 3G).
  c. These particles are further subjected to CTF refinement (per-particle defocus, per-micrograph astigmatism, beamtilt and high order aberrations) and 3D auto refinement, yielding four different conformations: IMS1 at 3.9 Å, IMS2 at 3.8 Å, IMS3 at 3.6 Å, and low-pH state at 3.4 Å, respectively (Figure 3D).
40. In the final step, to improve the density quality of the VP5 stalk region in the low-pH state, we use a method by combining the signal subtraction and focused 3D classification in Relion.
  a. Generate a soft mask which includes only the VP5 part. Subtract the density outside of the soft mask from low-pH state raw sub-particles 2D images by using Relion particle subtraction.
  b. Several rounds of focused 3D classification without orientation search with mask from step 40a are run (until no further improvement in the density map is observed) to sort out subtracted particles with good stalk density. In this step, 75,625 particles are finally selected.
  c. The corresponding original sub-particles without subtraction are selected in Relion and subsequently subjected to 3D auto-refinement, yielding a final reconstruction at 4.0 Å. This reconstruction is used to facilitate model building of VP5 stalk in low-pH state (Xia et al., 2021).

***Note:*** Most of the distal part of the VP5 stalk is unresolved in the sub-particle reconstruction even with further focused 3D classification, indicating the flexibility of the distal portion of the stalk. As the distal part of the VP5 stalk is important for membrane penetration, cryoET is used to investigate the interaction between VP5 and the liposomal membrane (described in the next section).

***Note:*** The resolutions of the cryoEM maps are estimated based on the gold-standard FSC = 0.143 criterion (Rosenthal and Henderson, 2003). The cryoEM maps were auto-sharpened by the *relion_postprocess* program in Relion (Scheres, 2012). The local resolution evaluations are determined by ResMap (Kucukelbir et al., 2014).

41. The atomic models are built according to the sharpened maps in Coot (Emsley and Cowtan, 2004) and refined by real space refinement in Phenix (Adams et al., 2010) (Figure 3H).
  a. Fit the anchoring domain structure of VP5 at pH 8.8 (PDB3J9E) (Zhang et al., 2016) into the low-pH state map (3.4 Å).
  b. Manually adjust the model in Coot according to the density map.
  c. Build the stalk of VP5 according to the low pH focus map (4 Å) with guidance from the structure of pH 8.8.
  d. Refine the model in real space according to the low-pH state map (3.4 Å) by Phenix (Adams et al., 2010) and validate the model by wwPDB validation server.
  e. For the model of VP5 in IMS2, the refined atomic model of low-pH state is docked into the cryoEM map first. The helices of unfurling domain in VP5 are built in Coot (Emsley and Cowtan, 2004) with guidance from the structure of pH 8.8.

### CryoET and subtomogram averaging

**Timing: 1 week**

**Figure 4:**
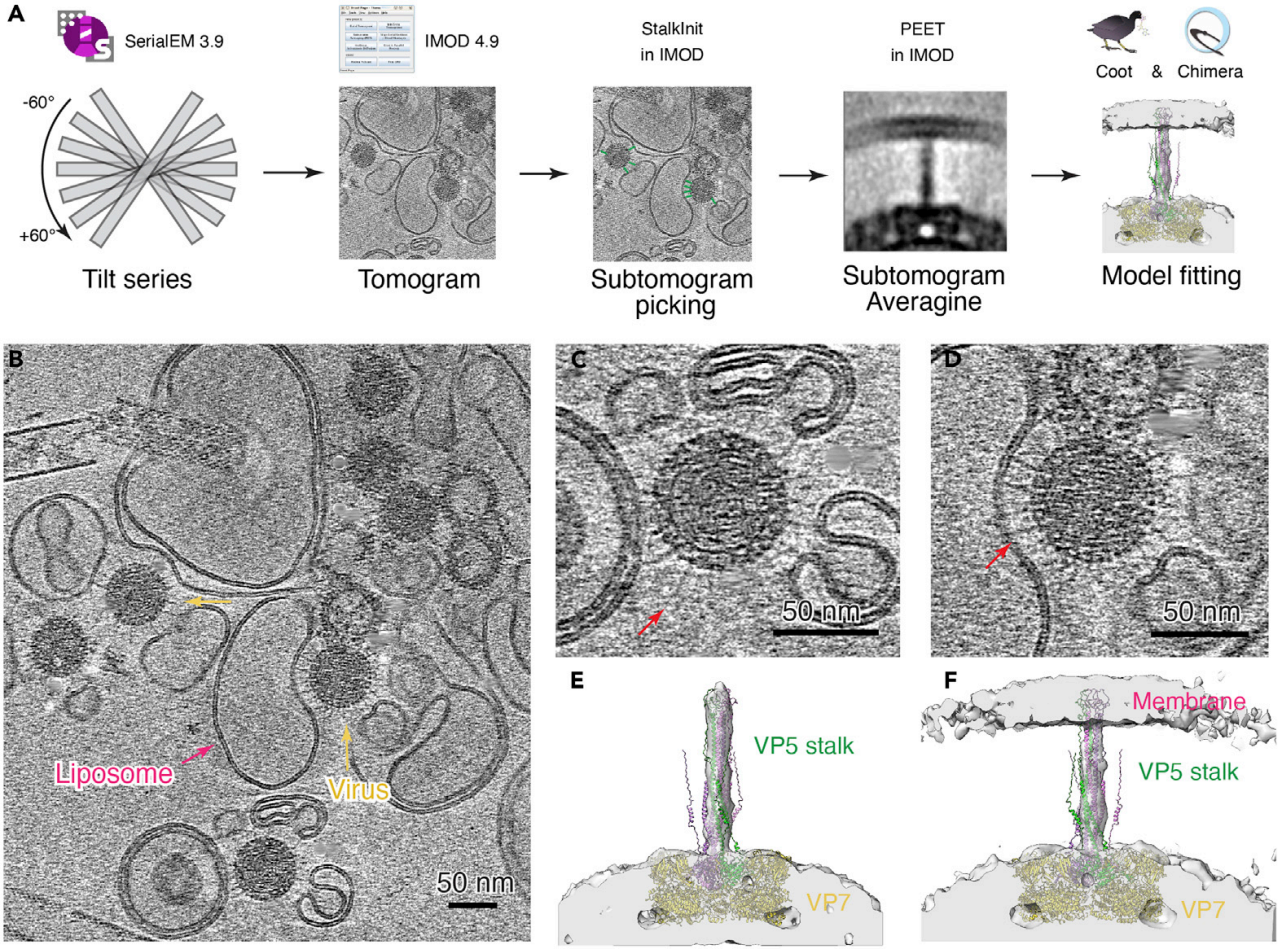
CryoET and subtomogramaverage (A) Workflow of cryoET and subtomogram average used in this paper. Programs used in each step are indicated. (B) A slice from a cryoET reconstruction of BTV particles incubated with liposomes in low-pH condition. Yellow and magenta arrows point to the virus and liposome, respectively. (C and D) Slices of cryoET reconstruction of BTV at low pH showing the membrane-free (C) and membrane-wrapped (D) BTV stalks. (E and F) Superimposition of the hypothetic model of full-length VP5trimer and surface view of the subtomogram average maps of the membrane-free stalk (E) and the membrane-wrapped stalk (F).

This section describes the detailed protocol for cryo-electron tomography and subtomogram averaging (Figure 4A). By using cryoET, we can observe the interactions between the distal part of VP5 stalk and the liposomal membrane.

42. Align the frames in each movie by MotionCor2 (Zhenget al., 2017). The images are binned 2× to a pixel size of 2.55 Å.
43. Generate a stack file including all the images from the same tilt series by using *newstack* program in IMOD (Figure 4B) (Kremer et al., 1996).
44. Estimate defocus values of aligned images in each tilt series by CTFFIND4 (Rohou and Grigorieff, 2015).
45. Tilt series are reconstructed with IMOD 4.9 software package (Kremer et al., 1996) by using selected gold beads. The estimated defocus value of each image is used as input for *ctfphaseflip.*

***Note:*** Two tomograms are generated by weighted back projection and simultaneous interactive reconstruction technique (SIRT) method, respectively. High-contrast SIRT tomograms are binned 4× (10.2 Å/pixel) by *binvol* program of IMOD and used for particle picking and display. For the low pH condition, a total number of 33 tomograms with good alignment and contrast are selected and further binned by 2× (5.1 Å/pixel) and 4× (10.2 Å/pixel) for further processing of subtomogram averaging.

46. Manually pick particles of VP5 stalks in IMOD. Each particle is represented by 2 points in a contour, with the first one for the capsid-proximal and the second one for the capsid-distal end of the stalk protruding from the virus capsid. Totals of 107 and 100 particles are picked for the membrane-free stalk and membrane-wrapped stalk, respectively.
47. Sub-volume averaging is performed in PEET software (Heumann et al., 2011; Nicastroet al., 2006) and 4× binned tomograms are used first. Initial orientations of particles are generated from the manually picked particles by using *stalkInit.*
48. Perform angular search with a cylindrical mask in PEET. The volume averaged without search is used as initial model. Search ranges along x and z axes are limited to 15 degrees while search range along y axis is 360 degrees.
49. The particles from step 48 are symmetrized to C3 along the particle Y axis by using *ModifyMotiveList* program in IMOD.
50. Align the symmetrized particles with a finer range till converge. Search ranges along x and z axes are limited to 9 degrees while search range along y axis is 120 degrees. The search ranges are decreased each round.
51. The refinement is then upgraded to 2× binned tomograms with model files and corresponding motive list files resampled from 4× binning to 2× binning. Run alignment again until convergence (Figures 4C–4F).
52. Calculate the resolutions of the subtomogram average by *calcFSC* in PEET(Nicastroet al., 2006). The resolution of membrane-free stalk and membrane-wrapped stalk is 24 Å and 27 Å, respectively, based on the 0.143 FSCcriterion (Xia et al., 2021).

***Alternatives:*** Relion 4.0 with subtomogram averaging has been released recently. To improve the resolution, shift and rotation parameters of particles from alignment in PEET can be imported into Relion for further refinement.

## Expected outcomes

Following this protocol, we can obtain several high-resolution structures of BTV in low pH condition, as well as subtomogram averages showing the interaction between the BTV stalk and the liposomal membrane (Xia et al., 2021). Typically, the concentration of BTV viruses obtained from 10 T175 flasks of cells is enough for cryoEM grid preparation (10 grids from 20 μL of virus with more than 5 particles in each cryoEM image). By collecting about four thousand micrographs for single-particle reconstruction, BTV structure at low pH can be reconstructed to atomic resolution by using the sub-particle method (Figure 3). From cryoET analysis, we can obtain high-quality tomograms showing the interaction between VP5 stalks and the liposomal membrane (Figure 4). Subtomogram averaging will lead to low resolution (20–30 Å) maps of VP5 stalk inserted into the membrane. If this protocol is applied to the studies of other non-enveloped viruses besides BTV, similar results can also be expected with minor modifications of the conditions that induce virus conformational changes.

## Limitations

This protocol has been successfully used in the study of BTV membrane penetration. However, some limitations still exist with this protocol. Due to the flexibility of the VP5 stalk, the distal portion of the stalk is largely invisible in the high-resolution maps from single-particle reconstruction even after focused classification. In the subtomogram averaging procedure, since the membrane-wrapped VP5 stalks are not present in every collected tomogram, it will be difficult to get a large number of particles for high-resolution reconstruction. As such, identification of residues which interact with the membrane from a low-resolution map would be very challenging.

## Troubleshooting

### Problem 1

Concentration of the purified virus is not high enough for cryoEM grid preparation.

### Potential solution

There are several possible reasons for this problem. First, the BHK-21 cells are not maintained well. Second, the timing for harvesting the infected cells has not been optimized. Third, most of the virus aggregates and goes into the pellet in step 11. To solve these problems, make sure to maintain the cells as described in this protocol. Try several different time points to harvest the infected cells in step 3 to find the optimal harvest time with the highest yield. If virus concentration is still not sufficient after above troubleshooting, try 20–30 flasks of cells instead of 10 for each purification.

### Problem 2

No conformational changes of BTV are observed by cryoEM.

### Potential solution

Use 200 mM sodium citrate buffer to change the sample pH instead of 100 mM in step 21. Change the incubation time from 30s to 1 min at step 24.

### Problem 3

Cannot achieve high-resolution reconstruction of BTV by single-particle reconstruction.

### Potential solution

First, make sure that the microscope is well aligned during data collection. This is a prerequisite of high-resolution reconstruction. To assess whether the collected images are of high quality, the Fourier transform of the images from step 34 should be checked. If the thon rings of most images reach a resolution beyond 5 Å, this indicates high quality of the data set. Thick ice of the cryoEM grid may also hinder high-resolution reconstruction. To solve this problem, select squares with thinner ice for data collection in step 28.

### Problem 4

Cannot capture images of BTV interacting with liposome.

### Potential solution

Before collecting tilt series at a target position, take an image in preview mode at this location with exposure time set to 0.5 s and camera parameters the same as in record mode. If the preview image has no virus particles interacting with liposomes, skip this target position.

Another possible reason for this problem is that the liposome concentration for cryoEM grid preparation is not high enough. To solve this problem, increase the concentration of liposomes by reducing the volume of the liposome buffer used in step 17.

## Resource availability

### Lead contact

Further information and requests for resources and reagents should be directed to and will be fulfilled by the lead contact, Dr. Z. Hong Zhou (Hong.Zhou@UCLA.edu).

### Materials availability

All unique reagents generated in this study will be shared by the lead contact upon request.

## Data Availability

This paper does not report original codes. The cryoEM density maps have been deposited in the Electron Microscopy Data Bank (EMDB): IMS1 (EMD-24686), IMS2 (EMD-24685), IMS3 (EMD-24687), and low-pH state (EMD-24684). The corresponding models of VP5 have been deposited in the Protein Data Bank (PDB): IMS2 (PDB: 7RTO) and low-pH state (PDB: 7RTN).
